# Screening Ultrasonography in a Basic Emergency Service for Suspected Acute Appendicitis: A Case Report

**DOI:** 10.7759/cureus.115364

**Published:** 2026-08-28

**Authors:** Sergio Miravent, Carmen J Bermejo, Carolina A Guerreiro, Magda M Silva, Manuel D Lobo, Rui P Almeida

**Affiliations:** 1 Basic Emergency Service of Vila Real de Santo António, Unidade Local de Saúde (ULS) Algarve, Vila Real de Santo António, PRT; 2 Medical Imaging and Radiotherapy, School of Health, University of Algarve, Faro, PRT; 3 Medical Dosimetry, Joaquim Chaves Saúde, Lisbon, PRT; 4 Radiology, Northeast Local Health Unit, Basic Emergency Service of Mogadouro, Mogadouro, PRT; 5 Health, Medicine and Life Sciences, University of Luxembourg, Esch-sur-Alzette, LUX

**Keywords:** appendicitis, case reports, emergency department, hospital referral, triage, ultrasonography

## Abstract

Suspected acute appendicitis is a frequent clinical scenario in Basic Emergency Service (BES), particularly among patients presenting with acute abdominal pain. However, its clinical presentation is not always straightforward. In the early stages, symptoms may be nonspecific and overlap with other causes of abdominal pain, while the limited diagnostic resources available in peripheral emergency units may reduce the certainty of the initial clinical assessment. We present the case of a 22-year-old male who presented to a BES with acute abdominal pain associated with vomiting and food intolerance, with pain predominantly located in the lower abdominal quadrants and more evident on the right side. In the setting of a clinical presentation suggestive of acute appendicitis, screening ultrasonography was not intended to establish a definitive diagnosis but rather to add objective clinical information to the initial assessment and support a more reasoned decision regarding patient management. The identification of sonographic findings compatible with possible acute appendicitis strengthened the clinical suspicion and contributed to justifying central hospital referral. This case supports the potential role of screening ultrasonography as a complementary tool in peripheral or prehospital emergency settings, particularly when clinical findings are suggestive but insufficient to guide patient management with confidence. In such contexts, ultrasonography may help support decisions ranging from local treatment and discharge to hospital referral or follow-up by the patient’s family physician.

## Introduction

Acute abdominal pain represents a substantial proportion of ED visits, accounting for approximately 5.8% of all emergency presentations in a large urban ED study [1]. In patients presenting with lower abdominal pain, suspected acute appendicitis remains a frequent and clinically relevant diagnostic consideration; however, its detection may be challenging, particularly in early or atypical presentations [2]. This diagnostic uncertainty is clinically relevant, as a proportion of patients with appendicitis may not be diagnosed during the initial ED assessment, particularly when presenting with undifferentiated or overlapping symptoms; some may require reassessment or return to care before the condition is recognized as likely requiring referral for urgent surgical evaluation [3,4].

Clinical findings may overlap with a broad spectrum of intra-abdominal conditions, including gastrointestinal, urinary, gynecological, and vascular disorders [5]. Extra-abdominal conditions, including thoracic and musculoskeletal disorders, may also mimic abdominal pathology and further complicate the initial assessment [6]. Therefore, in peripheral emergency units such as Basic Emergency Service (BES), where access to advanced laboratory testing or cross-sectional imaging may be limited, the initial orientation of patients with suspected acute appendicitis can be particularly challenging. In this context, when performed by appropriately trained personnel, screening ultrasonography may provide additional objective information, not as a definitive diagnostic tool, but as a complementary method to support clinical reasoning and strengthen the justification for patient orientation.

Classically, acute appendicitis may present with vague periumbilical pain, anorexia, nausea or vomiting, migration of pain to the right lower quadrant, and low-grade fever [7]. On physical examination, right lower quadrant tenderness, guarding, and rebound tenderness, including a positive Blumberg sign [8], may further support the suspicion of peritoneal irritation, although their absence does not exclude acute appendicitis. However, this typical clinical sequence is not always present, and laboratory findings may also vary. Leukocytosis and neutrophilia can support the clinical suspicion, but normal or only mildly abnormal inflammatory markers do not exclude acute appendicitis, particularly in early or atypical presentations [9]. White blood cell count and CRP should therefore be interpreted within the overall clinical context rather than as isolated diagnostic criteria. In the BES setting described in this case, CRP testing was not available, further increasing the relevance of clinical reassessment and screening ultrasonography as complementary tools to support patient orientation.

Regarding sonographic findings, features that should raise the suspicion of ongoing acute appendicitis include the visualization of a blind-ending, aperistaltic, non-compressible tubular structure arising from the cecum, usually with an outer diameter greater than 6 mm [10]. Additional supportive findings include appendiceal wall thickening, loss or alteration of mural stratification, focal tenderness during graded compression, increased echogenicity of the periappendiceal fat, appendicolith with posterior acoustic shadowing, periappendiceal free fluid or collection, and increased mural or periappendiceal vascularity on color Doppler [11]. These findings should be interpreted in conjunction with the clinical context, as screening ultrasonography may support the suspicion of acute appendicitis but should not be considered in isolation from clinical assessment and patient evolution.

## Case presentation

A 22-year-old male presented to a BES with acute abdominal pain that had started during the night, waking him at approximately 00:30. He reported one episode of vomiting and subsequent food intolerance. He denied fever, diarrhea, previous similar episodes, chronic medication, or known drug allergies.

On physical examination, the pain was predominantly located in the lower abdominal quadrants, more evident on the right side, and worsened with palpation. A mild peritoneal reaction on decompression was documented, corresponding to a positive Blumberg sign. Initial laboratory assessment showed leukocytosis with neutrophilia, supporting the suspicion of an acute inflammatory abdominal process.

Given the clinical suspicion, abdominal and pelvic screening ultrasonography was requested by the doctor on duty at the BES and performed by a radiographer/sonographer with formal academic training in ultrasonography as a complementary examination to support the physician’s clinical triage decision. The examination identified in the right lower flank a non-peristaltic, layered tubular structure compatible with an enlarged appendix, measuring more than 6 mm, associated with periappendiceal fluid (Video 1).

**Video 1:** Long-axis view of a tubular structure compatible with the appendix. Right lower quadrant screening ultrasonography showing an apparently enlarged, aperistaltic, non-compressible tubular structure compatible with the appendix, with adjacent periappendiceal free fluid and possible subtle echogenic change in the posterior periappendiceal fat. Overall, these findings were compatible with a local inflammatory process. The asterisk indicates periappendiceal free fluid near the appendiceal tip.

Focal tenderness during the ultrasound examination was also described. No additional relevant abnormalities were identified in the remaining assessed abdominal organs or peritoneal recesses.

**Figure 1:**
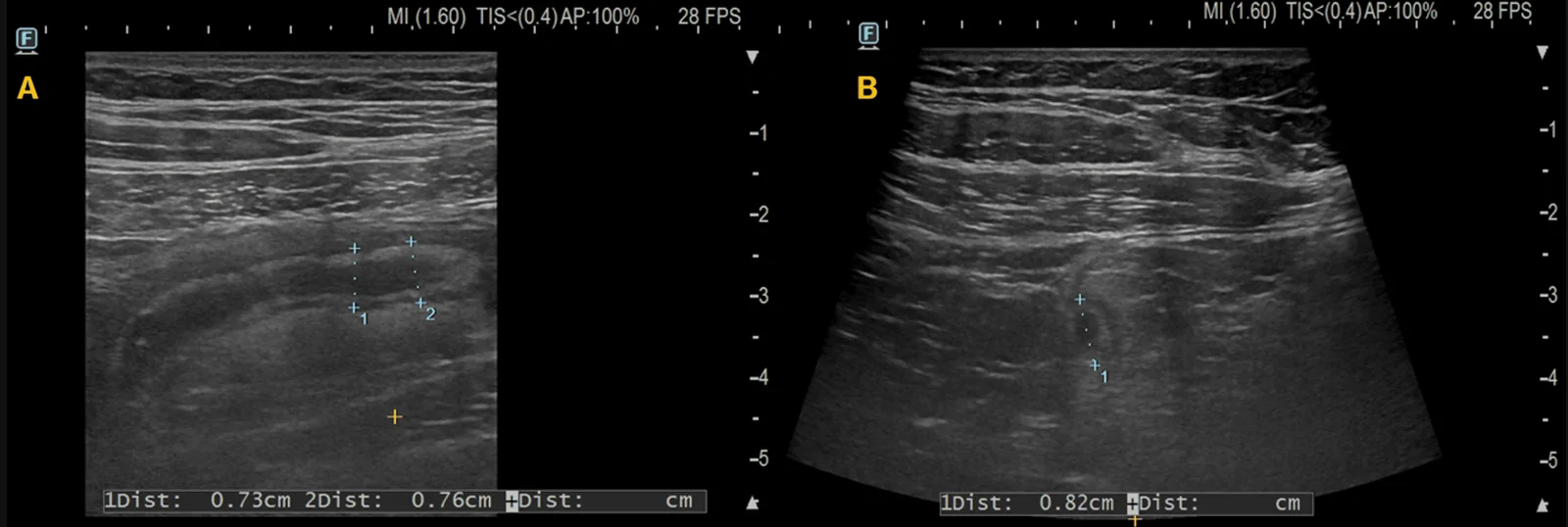
(A, B) Biplanar outer-diameter measurement of a tubular structure compatible with the appendix. Still images representing longitudinal and transverse views demonstrate appendiceal outer diameters of approximately 7.6 mm and 8.2 mm, respectively. Adjacent periappendiceal free fluid and a subtle increase in echogenicity of the posterior periappendiceal fat are also seen.

Because a sector transducer was available, the suspected appendiceal structure was assessed in two orthogonal planes (Video 2) and with graded compression (Video 3). Still images documenting the outer-diameter measurements are presented in Figure 1. A limitation of the sonographic assessment was the absence of documented color Doppler evaluation of the appendix.

**Video 2:** Biplanar assessment of the appendix using a sector transducer. (A, B) Longitudinal and transverse planes of the right lower quadrant during screening ultrasonography performed with a sector transducer, demonstrating an apparently enlarged, aperistaltic tubular structure compatible with the appendix. Adjacent periappendiceal free fluid and subtle increase in echogenicity of the posterior periappendiceal fat are also observed, overall compatible with a local inflammatory process. F.F., free fluid.

**Video 3:** Dynamic graded-compression assessment of a tubular structure compatible with the appendix. During graded compression, the tubular structure compatible with the appendix remained non-compressible despite repeated transducer pressure, without significant variation in its outer diameter. The maneuver was tolerated by the patient but elicited transient focal discomfort.

These findings were considered compatible with a possible acute inflammatory process and strengthened the justification for hospital referral.

At the referral hospital, an abdominopelvic CT scan with IV contrast was performed for further diagnostic clarification.

A swollen ileocecal appendix with increased mural enhancement is identified, located in the right paracolic gutter, measuring 10 mm in caliber, associated with densification of the adjacent fat and a thin layer of fluid, compatible with acute appendicitis. No organized collections or pneumoperitoneum are identified, namely findings suggesting appendiceal rupture. There is no distension of intestinal loops. The liver, gallbladder, pancreas, adrenal glands, and spleen show no relevant abnormalities. The kidneys are in normal topography, with preserved dimensions and enhancement, without signs of obstructive uropathy.

The CT findings, illustrated in Video 4, supported the diagnosis of acute appendicitis without imaging signs of organized collection, pneumoperitoneum, or appendiceal rupture. The patient was subsequently evaluated by the surgical team, and acute appendicitis was assumed as the working diagnosis. At admission to the referral hospital, laboratory testing showed leukocytosis (18.8 × 10⁹/L), marked neutrophilia (83.5%; absolute neutrophil count 15.7 × 10⁹/L), and an elevated CRP level of 53 mg/L. The patient underwent surgical treatment, with no reported intraoperative or immediate postoperative complications. The histological report described an appendectomy specimen measuring 7.8 cm in length, with a diameter ranging from 0.8 to 1.1 cm, and an irregular, violaceous serosa. The final histological diagnosis was acute phlegmonous appendicitis. By discharge, the leukocyte and neutrophil counts had normalized, while CRP had decreased to 31 mg/L, although remaining above the reference range.

**Video 4:** (A-C) Abdominopelvic CT cine-loops in suspected acute appendicitis. The region of interest is highlighted by a white dotted ellipse in all three sequences. (A) Axial non-contrast sequence through the right lower quadrant, allowing identification of the swollen appendix in the ileocecal/right paracolic region before IV contrast administration. (B) Axial contrast-enhanced sequence showing inflammatory changes in the same region, with adjacent fat densification and minimal periappendiceal fluid, corresponding to the reported location of the swollen ileocecal appendix. (C) Coronal contrast-enhanced reformatted sequence showing the same region of interest and its anatomical relationship with adjacent abdominal structures. Overall, the CT findings are compatible with acute appendicitis, with no evident organized collection or pneumoperitoneum. G.B., gallbladder; P.V., portal vein

## Discussion

This case illustrates the role of screening ultrasonography in a BES when acute appendicitis is clinically suspected, but the available diagnostic resources are limited. The aim of the examination was not to establish a definitive diagnosis but to identify possible sonographic signs that could strengthen the clinical suspicion and support a safer and more justified patient orientation.

Screening ultrasonography has important limitations that should be acknowledged. It is operator-dependent, and visualization of the appendix may be affected by patient body habitus, overlying bowel gas, pain-limited graded compression, local equipment or transducer availability, and the variable anatomical position of the appendix. In particular, retrocecal, retroileal, or deep pelvic appendiceal positions may make sonographic identification difficult or even impossible in some patients. Therefore, a negative or inconclusive screening ultrasound examination should not be used to exclude acute appendicitis when clinical suspicion persists.

In this patient, the clinical presentation raised suspicion of acute appendicitis. Screening ultrasonography identified findings compatible with this condition, including an enlarged, non-peristaltic, and non-compressible appendix and periappendiceal fluid. These findings provided sonographic support for an appendiceal/periappendiceal inflammatory process [12,13].

At the referral hospital, CT confirmed findings compatible with acute appendicitis, describing a swollen ileocecal appendix with increased mural enhancement, adjacent fat densification, and a thin layer of fluid, without organized collection, pneumoperitoneum, or signs of appendiceal rupture. The case therefore followed a coherent diagnostic and therapeutic sequence: clinical suspicion, screening ultrasonography, justified referral, CT confirmation, surgical evaluation, and operative treatment. In this case, surgery was performed without reported complications, and the patient had a short hospital course, with approximately 2.5 days from ED admission through operative management to discharge. By discharge, hematological parameters had normalized and CRP had decreased substantially, although it remained above the reference range, supporting a favorable uncomplicated postoperative course.

Early orientation is particularly relevant because delayed diagnosis may allow progression to complicated appendicitis, which is associated with greater morbidity, increased healthcare costs, and longer hospital stay [14,15]. In this context, screening ultrasonography may contribute to a favorable clinical cascade by supporting earlier referral and timely surgical management, while still respecting the role of CT and surgical assessment as part of the definitive diagnostic pathway.

Histopathological confirmation of acute phlegmonous appendicitis completed the diagnostic pathway, allowing correlation between the clinical presentation, screening ultrasound findings, CT assessment, surgical management, and the final anatomical diagnosis.

## Conclusions

This case illustrates the value of screening ultrasonography in the initial assessment of suspected acute appendicitis in a BES. In a clinically suggestive but not fully specific presentation, the identification of an enlarged, non-compressible appendix associated with periappendiceal fluid provided objective imaging findings that strengthened the clinical suspicion and enabled the BES physician to make a more confident and well-supported referral decision. CT subsequently confirmed acute appendicitis without signs of perforation or organized collection, followed by surgical treatment and histological confirmation of acute phlegmonous appendicitis. Screening ultrasonography should not be regarded as a definitive diagnostic method in this setting but as a complementary examination that, when integrated with clinical assessment and performed by a trained sonographer, may provide additional objective information to support appropriate hospital referral based on locally acquired sonographic and clinical evidence.
